# Development of a Microfluidic Viscometer for Non-Newtonian Blood Analog Fluid Analysis

**DOI:** 10.3390/bioengineering11121298

**Published:** 2024-12-20

**Authors:** Yii-Nuoh Chang, Da-Jeng Yao

**Affiliations:** 1 Institute of NanoEngineering and MicroSystems, National Tsing Hua University, Hsinchu City 300, Taiwan; enochchang070@gmail.com; 2 Department of Power Mechanical Engineering, National Tsing Hua University, Hsinchu City 300, Taiwan

**Keywords:** non-Newtonian fluid, microfluidic device, blood behavior flow

## Abstract

The incidence of stroke is on the rise globally. This affects one in every four individuals each year, underscoring the urgent need for early warning and prevention systems. The existing research highlights the significance of monitoring blood viscosity in stroke risk evaluations. However, the current methods lack the precision to measure viscosity under low shear rate conditions (<100 s⁻¹), which are observed during pulsatility flow. This study addresses this gap by introducing a novel microfluidic platform designed to measure blood viscosity with high precision under pulsatility flow conditions. The systolic blood viscosity (SBV) and diastolic blood viscosity (DBV) can be differentiated and evaluated by using this system. The non-Newtonian behavior of blood is captured across specific shear rate conditions. The platform employs a meticulously designed microarray to simulate the variations in blood viscosity during pulsation within blood vessels.The results demonstrate an impressive accuracy of 95% and excellent reproducibility when compared to traditional viscometers and rheometers and are within the human blood viscosity range of 1–10 cP. This monitoring system holds promise as a valuable addition to stroke risk evaluation methods, with the potential to enhance prediction accuracy.

## 1. Introduction

In recent years, the integration of advanced biomedical knowledge and cutting-edge technologies has significantly improved the accuracy and scope of stroke prediction models [1,2,3]. Stroke, as one of the leading causes of disability and mortality worldwide, often occurs without warning, leaving profound impacts on individuals, families, and society [4,5,6]. It happens when the blood supply to the brain is suddenly interrupted or when a blood vessel in the brain ruptures, resulting in the death of brain cells. A critical factor in these advancements is the consideration of blood viscosity (BV), an essential parameter reflecting the thickness and flow properties of blood [7,8,9]. Emerging studies have revealed a complex relationship between abnormal BV and stroke risk [10,11,12]. High blood viscosity increases the risk of thromboembolism and is a significant contributing factor to cardiovascular diseases. These studies have demonstrated a correlation between blood viscosity and ischemic stroke [13,14,15]. The factors influencing blood viscosity are multifaceted, including blood sugar levels, red blood cell count, and thrombosis [16,17,18,19]. Accurate measurement of the relationship between blood viscosity and stroke is essential. Within 24 h of acute ischemic stroke onset, arterial pulsatility is closely associated with blood viscosity [14]. An appropriate physical measurement model should evaluate stroke risk by capturing the non-Newtonian properties of blood under varying shear rate conditions.

The physical parameters of blood flow, shear rate, and shear stress were identified as early as the 1970s and subsequently investigated for their potential impact on arterial thrombus formation [20]. In normal vascular flow, when arterial diameter narrows (stenosis) while maintaining a constant blood flow rate, wall shear rate and shear stress increase proportionally to the inverse cube of the vessel diameter [21,22]. In previous studies and experiments on factors related to blood viscosity, rotational viscometers were used to observe the non-Newtonian properties of blood [23,24,25,26]. However, the testing conditions of traditional rotational viscometers and rheometers are poorly suited for biological experiments. These instruments are limited by their ability to reliably measure the lowest torque, particularly when acquiring shear viscosity data under low shear rate conditions [27]. As a result, microfluidic platforms have been developed to study blood properties [28,29,30]. For instance, a parallel laminar flow microchip has been introduced for measuring the viscosity of Newtonian fluids [31,32,33]. The parallel laminar flow measurement model is a powerful method for achieving high-precision viscosity detection. In this study, we utilized specific microgeometry to simulate systolic blood viscosity (SBV) and diastolic blood viscosity (DBV) for measuring the blood viscosity characteristics under arterial pulsatile flow conditions [14]. This system offers an in vitro evaluation framework to observe blood viscosity variations, particularly at specific vessel wall locations under shear rate conditions, providing a potential basis for assessing stroke risk factors. The feasibility of quantifying blood viscosity will aid clinical research in establishing viscosity thresholds in prediction platforms, enabling the evaluation of individual stroke risk.

## 2. Materials and Methods

### 2.1. Parallel Laminar Flow Microchip System

**Figure 1:**
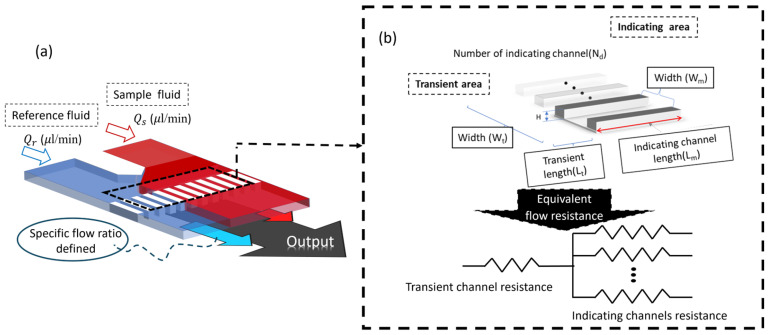
(**a**) Demonstrate the principles and operational concepts of the microfluidic platform. (**b**) Demonstrate the physical symbolic representation of the functional resistance in the microfluidic chip.

The experimental setup involves a two-phase fluid system where both the experimental sample and reference fluid are simultaneously injected into the chip, shown in Figure 1a. The flow input condition is provided by an input system which is consist of two syringe pumps (KD scientific, Holliston, MA, USA) and two flowmeters (FLU-M, FLUIGENT, Le Kremlin-Bicêtre, France). The flow observation is relying on the inverted microscopy (CKX53, Olympus, Tokyo, Japan). The precision of the fluid control system is pivotal as any error directly impacts the measurement accuracy of the experimental platform. Consequently, the meticulous construction and rigorous verification of the system play a crucial role in ensuring the reliability and accuracy of the experimental results. An alignment standard relies on the vibration viscometer (VM-10L, SEKONIC, Tokyo, Japan) and rheometer (AR2000, TA Instrument, Wilmslow, UK).

### 2.2. Sources of Newtonian and Non-Newtonian Flow Samples in the Experiments

The shear-thinning and viscoelastic properties of blood in viscoelastic flow are closely related to its microscopic structure [34]. Aqueous xanthan gum solutions have been proven to be one of the more successful blood analog fluids [35]. We prepared xanthan gum (X0048, MP SINGAPORE, Singapore) solution as our non-Newtonian samples. Concentrated glycerol solutions (Glycerol, Merck, Rockville, MD, USA) and simulated blood dry (SIMULATED BLOOD, VATA, Canby, OR, USA) were used as Newtonian control fluids with varying viscosities (Table 1).

### 2.3. Chip Design and Fabrication

**Figure 2:**
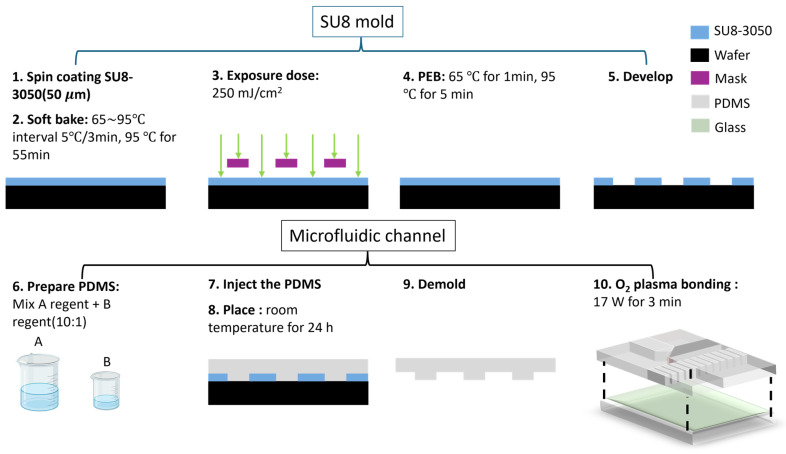
A demonstration of the microfluidic chip fabrication flow.

A polydimethylsiloxane (PDMS) Sylgard 184 silicone elastomer base and Sylgard 184 elastomer curing agent were purchased from Dow Corning Corporation (Midland, MI USA). The silicone mold is fabricated based on conventional photolithography techniques, as shown in Figure 2. The fabrication process begins with spin-coating SU-8-3050 (SU-8 3050, Kayaku Advanced Materials, Westborough, MA, USA) photoresist onto the substrate to achieve a uniform thickness of 50 μm. The spin-coating process ensures precise thickness control, which is critical for subsequent patterning.

Following the coating, a soft bake was performed to remove residual solvents and stabilize the photoresist layer. The soft bake consisted of a multi-step ramp, which starts at 65 °C, and a temperature increase in 5 °C intervals every 3 min until 95 °C. We maintained the 95 °C for 55 min. The coated and baked substrate was then exposed to ultraviolet (UV) light with an exposure dose of 250 mJ/cm^2^. This exposure initiates cross-linking within the photoresist, defining the desired pattern in the exposed regions.

A post-exposure bake (PEB) was performed immediately after exposure to further promote cross-linking and improve the resist’s adhesion and mechanical properties. The PEB consists of two steps: heating at 65 °C for 1 min, followed by heating at 95 °C for 5 min. This two-step bake optimizes the resolution and profile of the patterned structures.

Finally, the development process was carried out to remove the unexposed regions of the SU-8-3050. The developer selectively dissolves the uncross-linked photoresist, leaving behind the desired high-resolution features.

The microfluidic chip was replica with a typical soft lithography technique. Polydimethylsiloxane (PDMS; Sylgard 184 A/B, Dow Corning, Corporation, Midland, MI, USA) was irrigated to the silicon mold with a typical mixing ratio of Sylgard 184 A and B. The mold was degassed in a vacuum desiccator until all air bubbles were removed, then cured in an isothermal oven for 24 h at room temperature. The PDMS slab was bonded to a flat glass slide thereafter via oxygen plasma treatment for 3 min, and then the microfluidic chip was placed under a hydraulic press to ensure complete sealing. The microfluidic chip consisted of 100 microarray channels, shown in Figure 1b; each channel is 50 μm in height, 100 to 250 μm in width, and 500 to 4500 μm in length. The microarray channels provided a specific shear rate design to simulate the systolic and diastolic blood flow conditions, which is a representation of the artery pulsatility flow in the vessel environment [14]. More experimental chips easily presented the viscosity value by counting the number of channels filled.

### 2.4. Principle of Viscosity Measurement

In viscosity measurement, a dual-phase laminar flow microfluidic chip was designed to measure the viscosity of fluids. As shown in Figure 1a, two fluids are simultaneously injected into the wafer. One fluid with a known viscosity is referred to as the reference fluid, while the other fluid, which has an unknown viscosity, is referred to as the sample fluid. The fluids flow parallelly to the measurement zone. The microfluidic chip is designed with two distinct functional areas: the transient area and the microarray area. The sample and reference fluids are injected into the transient area of the chip under specific flow rate conditions (Q_s_ and Q_r_). The fluid resistance of the microarray area (R_m_) was designed to be much larger than the transient area (R_t_), which was designed as R_m_ ≫ R_t_. The flow resistance follows Poiseuille’s law, as shown in Figure 1b.

The resistance of each channel is calculated using Equations (1) and (2).

(1) $$R_{t}=\frac{12\cdot \mu \cdot L_{t}}{w_{t}h^{3}}\cdot {\left(1-\frac{192}{\pi^{5}}\frac{h}{w}\sum_{n=1,3,5\ldots }^{\infty }tanh\;(\frac{n\pi \omega }{2h})\right)}^{-1}$$

(2) $$R_{m}=\frac{12\cdot \mu \cdot L_{m}}{w_{m}h^{3}N}\cdot {\left(1-\frac{192}{\pi^{5}}\frac{h}{w}\sum_{n=1,3,5\ldots }^{\infty }tanh\;(\frac{n\pi \omega }{2h})\right)}^{-1}$$

where R_t_ is the flow resistance of the transient area, R_m_ is the flow resistance of the microarray area, μ is the viscosity of the fluid that passes through the transient area, L_t_ and L_m_ are the lengths of the transient area and microarray area, w_t_ and w_m_ are the widths of the transient area and microarray area, N is the number of microarray channels, h is the height of the channel of the chip. Under the condition that the two-phase flow was fully developed, the reference and sample fluids can flow into the microarray channels with fully developed flow phenomena. The pressure variable is described as:

(3) $$∆P_{s}=Q_{s}\cdot R_{s}$$

(4) $$∆P_{r}=Q_{r}\cdot R_{r}$$

**Figure 3:**
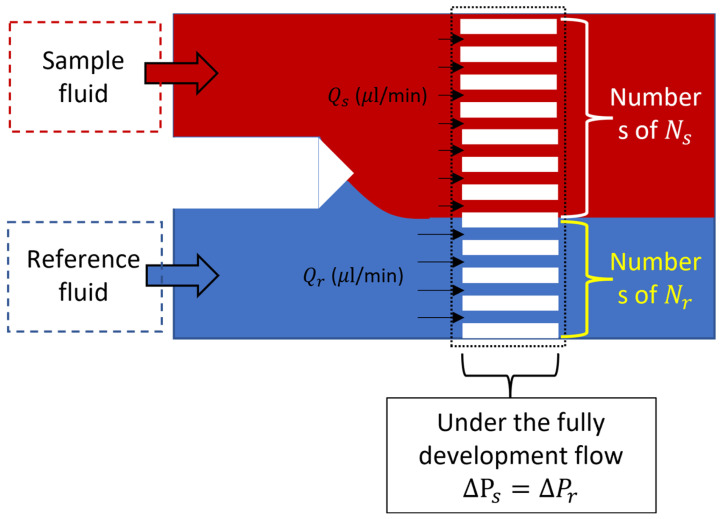
Demonstration of the flow-resistance circuit, which is expressed in parallel at the same pressure drop.

The pressure drops of each microarray channel of the sample and reference fluids are the same, and the fluids can meet the same pressure drop (ΔP_s_ = ΔP_r_), which is determined by the hydraulic pressure, as shown in Figure 3.

The pressure drop of the sample fluid and reference fluid in each microarray channel is identical, satisfying the condition of equal pressure drop (ΔP_s_ = ΔP_r_), which is determined by hydraulic pressure, as shown in Figure 3. Therefore, the relationship of viscosity is representing as:

(5) $$\mu_{s}=\frac{Q_{r}}{Q_{s}}\cdot \mu_{r}\cdot \frac{R_{s}}{R_{r}}$$

Variables in the relative concentrations of the reference fluid and sample fluid will affect the interface position of the two-phase flow. The expression method of the interface position can be simplified by the number of channels in the index flow channel area, so the calculation method of viscosity measurement can be simplified as:

(6) $$\mu_{s}=\frac{Q_{r}}{Q_{s}}\cdot \mu_{r}\cdot \frac{N_{s}}{N_{r}}$$

where the N_s_ and N_r_ are the sample and reference fluids of the number of microarray channels. The viscosity of the sample can be evaluated by Equation (6) based on observing the channel numbers on the microarray chip.

### 2.5. Microarray Design

In this experiment, different microarray designs were adopted to simulate the fluid behavior of blood transport in vessels, providing various shear rates. When non-Newtonian fluids pass through the microarray channels, blood viscosity changes. Defining different shear rates for various applications is crucial, particularly in blood viscosity measurement. The Yang research group proposed a standard flow channel to describe shear behavior [36,37].

(7) $$\dot{\gamma }=(\frac{6Q_{s}}{w_{m}h^{2}N_{s}})(\frac{\alpha }{\beta })$$

(8) $$\alpha =1-\frac{192}{\pi^{5}}\frac{h}{w_{m}}\sum_{n=1,3,5\ldots }^{\infty }\frac{1}{n^{5}}\tanh\;\left(\frac{n\pi w_{m}}{2h}\right)$$

(9) $$\beta =1-\frac{16}{\pi^{3}}\frac{h}{w_{m}}\sum_{n=1,3,5\ldots }^{\infty }\frac{1}{n^{3}}tanh\;(\frac{n\pi w_{m}}{2h})$$

The shear rate is defined by some factors, which are the delivery flow rate of the sample fluid, represented by *Q_s_*; the number of microarray channels filled with the sample fluid, which is represented by N_s_; the width of the indicator channel, represented by W_m_; and the flow channel height, represented by *h*. Followed by the description of the flow resistance and shear rate of the microarray, the high influence of factors is proposed as follows:

(10) $$\frac{R_{m}}{R_{t}}\propto \frac{L_{m}}{w_{m}}\times \frac{w_{t}}{L_{t}}$$

The parameter design of the physical description of the measurement applications is concluded in Equation (10). The $\frac{R_{m}}{R_{t}}$ is indicative of evaluating the situation of full development flow of non-Newtonian fluid. The $\frac{w_{t}}{w_{m}}$ and $\frac{L_{m}}{L_{t}}$ is the factor that we use to create the geometry model of blood vessels.

## 3. Results and Discussion

### 3.1. Physical Numerical Simulating Results

The analysis of theoretical and physical models involves the use of the versatile COMSOL Multiphysics 6.1 universal engineering CAE simulation software platform. The finite element method offers advantages in handling irregular shapes, complex physical couplings, and boundary conditions, making it highly suitable for studying the rheologi-cal states and flow fields of non-Newtonian fluids, such as blood, under various fluid boundary conditions. Given the complexity of blood as a non-Newtonian fluid, multiple models have been proposed in blood research, including the Carreau-Yasuda, Casson, and Power law models. These models aim to accurately predict the relationship between sample viscosity and shear rate. Among them, the Power law model was selected for its high degree of flexibility in describing fluid behavior within specific shear rate ranges. The parameters of this model can be precisely fitted using MATLAB, thereby improving the accuracy of blood viscosity predictions and enabling more accurate analyses of blood flow dynamics in the design of microarray chips.

**Figure 4:**
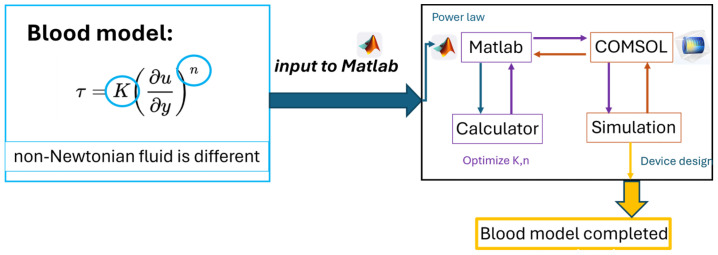
Numerical model of blood construct flow chart, which evaluated the K and n coefficients.

We used the Power law model to construct the blood behavior model and simulate the dual-phase flow of the sample and reference fluids. The interface distribution of the dual-phase fluids provides a method to verify the model’s accuracy through Equation (6). The blood viscosity database references the research of the E. Wells group [38], who contributed to the relationship between shear rate, hematocrit variability, and blood viscosity. The hematocrit-based blood viscosity model was constructed by fitting the Power law model, as shown in Figure 4.

**Figure 5:**
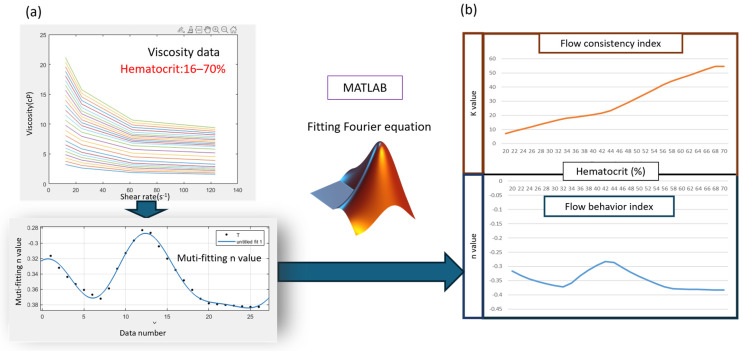
(**a**) Blood viscosity database with a range of hematocrit between 16 and 70% (**b**) Blood model fitting with power law for calculating K and n parameter values.

The R square value was used to evaluate the appropriate K and n values. Only results with an R square above 0.95 were adopted, as shown in Figure 5.

**Figure 6:**
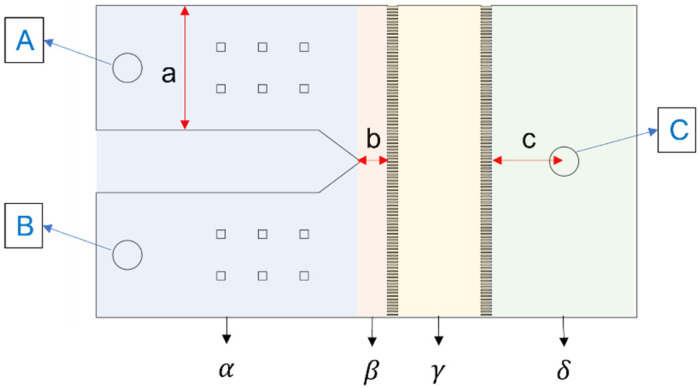
Schematic diagram of chip design parameters for numerical simulation experiments.

The hematocrit range for healthy adult males typically falls between 40–54%, while for females, it is 35–48%. In numerical simulations, a hematocrit value of 40% is selected as the standard for viscosity simulation samples. The microarray chip is systematically divided into distinct functional areas, including the flow channel entrance area, confluence area, measurement area, and flow channel exit area, as illustrated in Figure 6. Fluids are injected simultaneously at positions A and B. The fluid simulation parameters and boundary conditions are listed in Table 2. The α zone focuses on achieving fully developed flow; the β zone aims to provide sufficient coflowing length for the sample and reference fluids to achieve fully developed flow; the γ zone is primarily designed to optimize the spacing of microarray channels; and the δ zone is designated for fluid discharge. The functional design criteria are presented in Table 3. We proposed five different designs to observe the velocity streamline distribution of coflowing and fluid discharge. During the simulation, after fluid is injected into the channel, these functional areas are precisely defined to establish specific physical boundary conditions using COMSOL.

**Figure 7:**
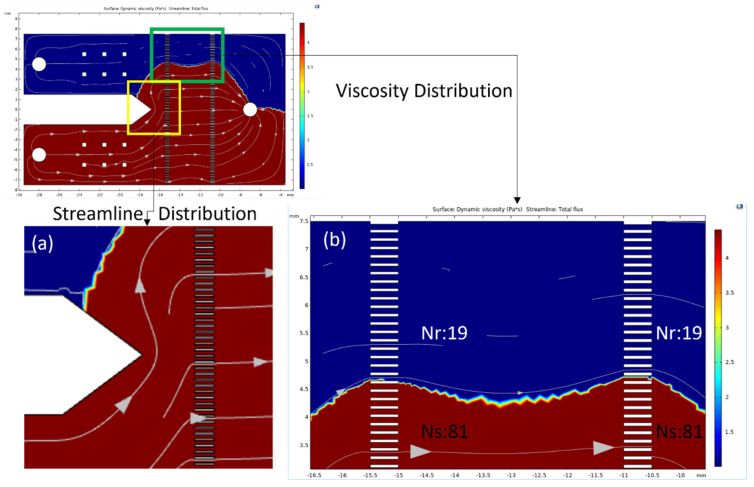
Numerical results of initial design by COMSOL. The red and blue regions are represented by sample and reference fluids. The streamline is represented by flux direction. (**a**) The streamline of sample fluid is squeezed in observations, and (**b**) the full channel number of sample and reference fluids shows the distribution region of the two fluids.

According to the simulation results, the fully developed flow of non-Newtonian fluids is estimated to be laminar [39]. When the fluid flows through the microarray channels, it remains in a fully developed flow state. Therefore, a 2D physical model is used for calculations to reduce the number of elements. Beyond changes in the flow field, the critical focus of observation lies in the interface between the sample and reference fluids. The streamlines in the parallel zone of the initial design show that the fluid is compressed in distribution and streamline slope, as illustrated in Figure 7a, indicating that the parallel zone in the initial design is insufficient in length. The interface between the two fluids is located in the parallel zone at the top of the chip, on the side of the reference fluid, as shown in Figure 7b. According to Equation (6), under identical flow rate conditions, the high-viscosity fluid occupies a larger area compared to the other fluid. The reference fluid and sample fluid occupy 19% and 81% of the microarray channel, respectively, consistent with the simulation results.

**Figure 8:**
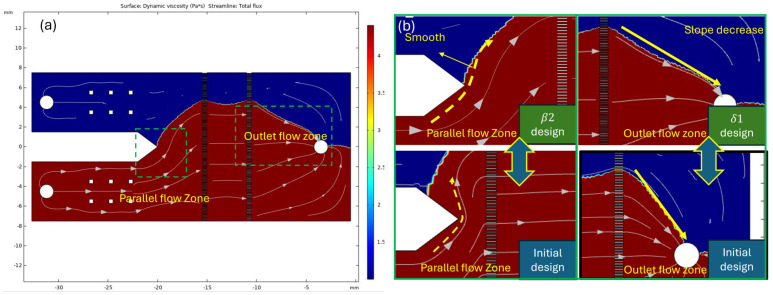
Numerical results of $\delta_{2}$ design in COMSOL. The red and blue regions are represented by sample and reference fluids. The streamline is represented by flux direction. (**a**) The parameters of “b” and “c” are revised. The adjustments provided a lesser variable for flux direction in the flow field. (**b**) The comparison of the initial and $\delta_{2}$ designs show that the $\delta_{2}$ design creates a more stabilized condition in chip design.

Based on the β_2_ and δ_1_ designs, parameters b and c contribute to the extension of the parallel flow zone and the outlet flow zone, respectively, achieving a low-variability and highly stable flow field. The specific parameter designs transitioning from β_2_ and δ_1_ to δ_2_ are illustrated in Figure 8a. In these designs, we observe that the fluid equivalent flow paths are connected in series, which are expected to provide stable and fully developed flow conditions within the measurement zone. The simulation results, shown in Figure 8b, indicate that the modified sections do not mutually affect each other. Each parameter effectively contributes to a stable flow field in its respective b and c regions. In the yellow-highlighted area, we specifically demonstrate smooth and non-distorted velocity streamlines. When the streamlines are not smooth and exhibit distortions, periodic oscillations may occur during operation, making the observation of the coflowing interface position difficult.

Based on the numerical results of the functional zone designs (as described in Figure 8), the δ_2_ design has been identified as the optimal solution for achieving smooth flow and minimizing compression phenomena in the confluence area, while extending these advantages to the outlet zone.

### 3.2. Implementation of Measurement Zone Design of Microarray Chip

**Figure 9:**
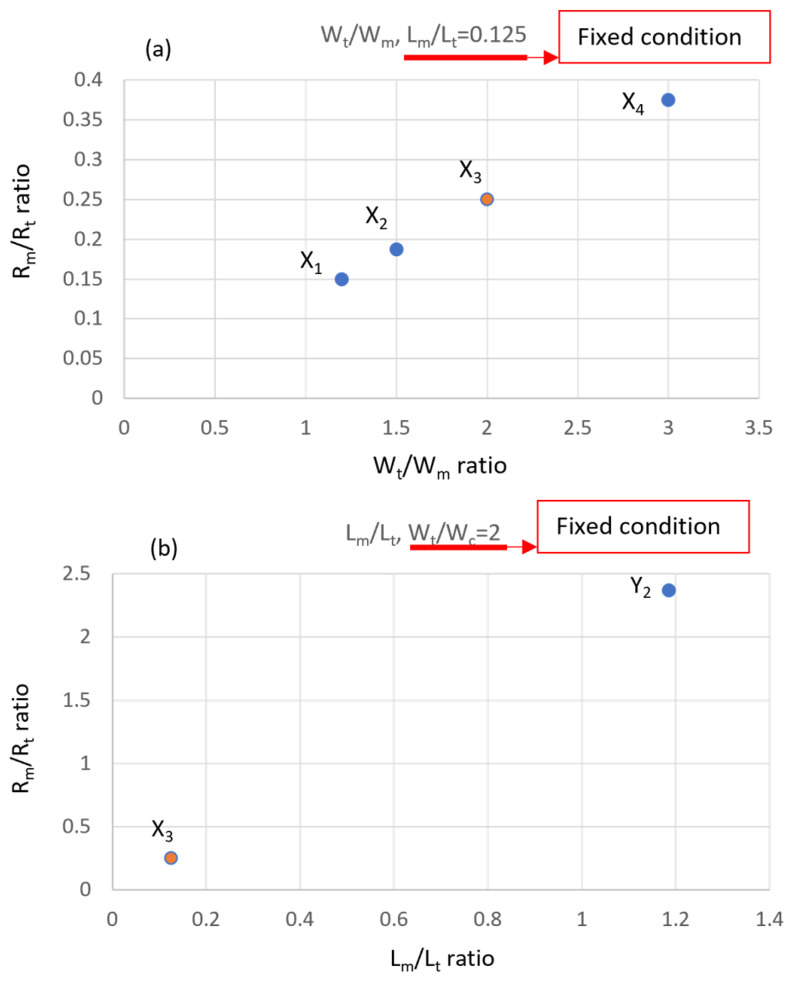
The geometry parameters of the width and length of the channel design at the measurement zone: (**a**) the designs X_1_ to X_4_ provided different $\frac{R_{m}}{R_{t}}$ values under $\frac{L_{m}}{L_{t}}$ = 0.125; (**b**) the designs X_3_ and Y_2_ provided different $\frac{R_{m}}{R_{t}}$ values under $\frac{w_{t}}{w_{m}}$ = 2.

Viscosity measurements were conducted at a room temperature of 26.0 ± 0.5 °C. The viscosity sample used artificial blood dye, representing a Newtonian fluid with a viscosity of 4.8 cP. Investigating the flow rate ratio is crucial for the accuracy of fluid dynamics and flow behavior in this experimental setup [33]. A flow rate ratio of 1:7 was used as an operational parameter to find the optimal solution. We proposed five chip designs based on the channel geometry of the measurement zone, derived from Equation (10). While keeping the length ratio constant, the fluid resistance ratio between the confluence zone and the measurement zone was altered by fine-tuning the channel width. Four different designs (X_1_ to X_4_) were proposed to simulate the fluid resistance conditions and shear rates of vessels with varying diameters, as shown in Figure 9a. Our focus was on the fluid resistance ratio from the parallel flow zone (R_t_) to the measurement zone (R_m_) under different width ratio conditions. A higher resistance ratio indicates stronger resistance and shear rate in the measurement zone, which affects the stability of the flow field. Additionally, channel length was fine-tuned under fixed width ratios to simulate the impact of different vessel lengths on fluid resistance. Designs X_3_ and Y_2_ provided different $\frac{R_{m}}{R_{t}}$ values under varying $\frac{w_{t}}{w_{m}}$ ratios, as illustrated in Figure 9b.

Through the above experimental designs, the conducted experiments allowed the interface position between the reference fluid and sample fluid to be determined via image analysis. Finally, the viscosity value can be converted using Equation (6). By using fluids with known viscosities, the experimental results can be used to evaluate measurement errors relative to the standard values. Based on the experimental designs in Figure 8, the results are shown in Figure 9.

**Figure 10:**
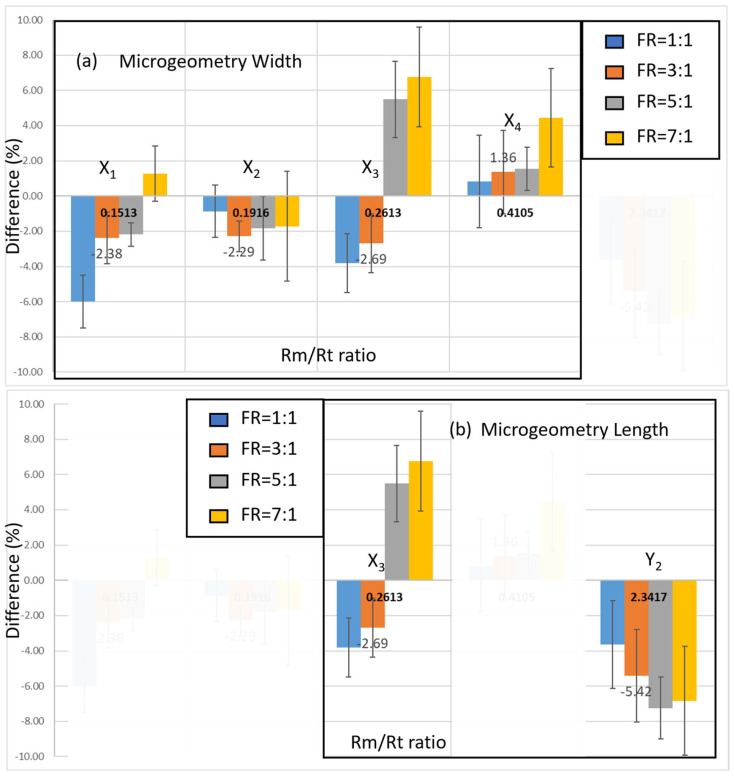
The calibration measurement results of the microfluidic chip: (**a**) consisting of the length design and observed accuracy model of measurements using the width variable; and (**b**) fixed width design and observed accuracy model trend using the length variable.

As shown in Figure 10a, the adjusted W_m_ design impacts the accuracy of viscosity measurements differently, with error ranges between −2.3% and 1.36% compared to the standard values. The model indicates that the X_2_ design parameters minimize errors under various flow rate ratios within the system. As illustrated in Figure 10b, adjustments to the microgeometry length increase system error from −2.5% to −5.4%, indicating an underestimation of the results.

A noteworthy finding in our study is the inverse relationship between flow rate ratio and measurement stability, where higher flow rate ratios correlate with decreased stability and accuracy. Conversely, when the flow rate ratio approaches 1, the measurement results exhibit greater accuracy and stability. Although the width of the microarray channels was considered in the design, distinguishing variations in the accuracy model remains challenging.

However, changes in the channel length design significantly impact the measurement data under different flow rate conditions, resulting in values lower than the actual viscosity. Therefore, we decided to adopt the X_2_ measurement zone design and low flow rate conditions for the implementation of both Newtonian and non-Newtonian fluid measurements.

### 3.3. Implementation of Newtonian Sample Viscosity Measurement

**Figure 11:**
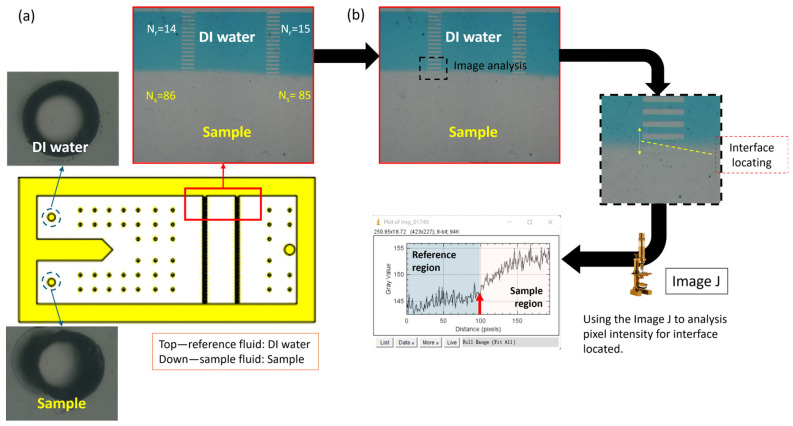
The method for implementation is illustrated above. (**a**) First, simultaneous injection of reference and sample fluids was conducted for 10 min, and the coflowing interface location was recorded. (**b**) Using the intensity analysis function in ImageJ, the fluid interface location was visualized.

The samples were prepared from aqueous glycerol solutions with concentrations ranging from 10 wt% to 40 wt%. Different flow rate ratio conditions were applied simultaneously to achieve high-precision measurements. The measurement results were further enhanced in viscosity resolution through image analysis software, as shown in Figure 11. Based on the images recorded by the system as shown in Figure 11a, we analyzed the image intensity to measure the interface position between the reference fluid and the sample fluid. The measurement range was determined based on the known structural length, fully covering the concentration dilution region, and outputting a grayscale intensity profile, as shown in Figure 11b. Compared to visual observation, this method allows for more accurate determination of the interface position.

**Figure 12:**
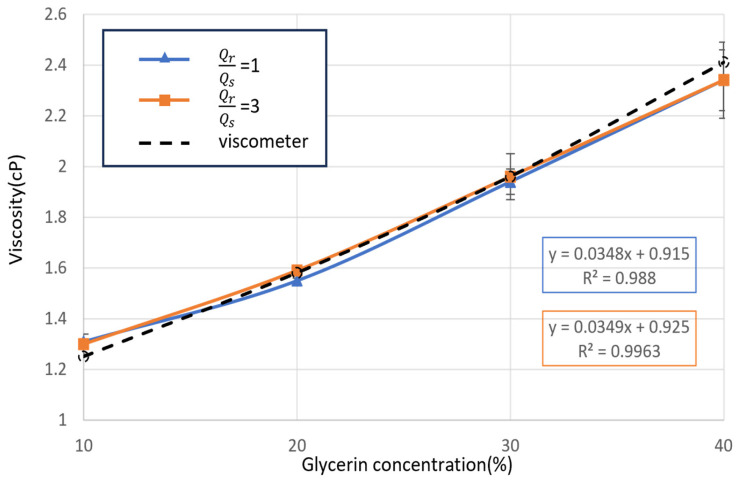
Implementation of the Newtonian fluid, 10–40% glycerin aqueous viscosity measurement.

The measurement results were compared with a traditional vibration viscometer, as shown in Figure 12. The viscosity measurements of Newtonian fluids revealed a highly similar trend between the traditional viscometer and the designed chip under various flow rate conditions. At flow rate ratios of 1 and 3, the maximum difference between the viscometer and the microarray chip measurements was less than 2%. In the Newtonian fluid tests, the operational flow rate conditions did not significantly affect the accuracy or precision of the viscosity measurements performed by the microarray chip.

### 3.4. Implementation of Non-Newtonian Sample Viscosity Measurement

**Figure 13:**
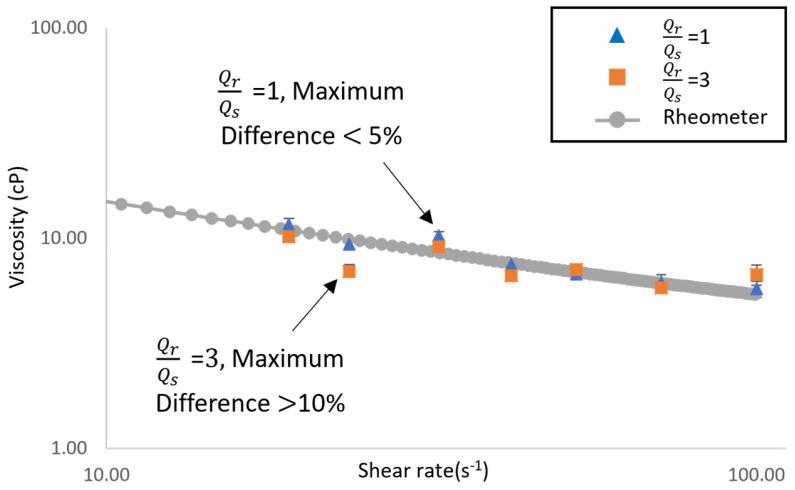
Implementation of non-Newtonian fluid, xanthan gum aqueous viscosity measurement. Three repeats for each measurement.

The blood sample (by using aqueous xanthan gum) is patient pending as a non-Newtonian measurement. The rheometer was used as an alignment standard for non-Newtonian fluid measurements. The viscosity measurement shown in Figure 13 can be used to compare the measurement difference between the rheometer’s and microarray chip’s results.

The measurement between the rheometer and microarray chip also showed a very high similarity in this non-Newtonian test. The measurement difference between the rheometer and microarray chip was lower than 5% in a flow rate of 1. The general blood flow velocity in the common carotid artery (CCA) for patients is 12.15 cm/s and 36.46 cm/s during diastole and systole [40]. Along with the CCA reference diameter of the vessel, this is used to calculate the shear rate [41]. The shear rate was determined as follows [42]:

(11) $$\dot{\gamma }=4\times \frac{V}{D}$$

where V and D are the fluid velocity and CCA diameter, respectively. Therefore, the shear rate started from 20 s^−1^ to 100 s^−1^ as DBV and SBV. The measurement result, with a flow ratio of 3, gave an unstable performance compared with the one using other conditions. However, the trends of SBV and DBV are high when conducting simulations with the rheometer. The operation flow rate condition significantly affects the accuracy and precision of microarray chip viscosity measurements in non-Newtonian fluid tests.

Our viscosity measurement model, specifically designed for non-Newtonian fluids, demonstrates significant advantages in accuracy, sample efficiency, and applicability under biologically relevant conditions. Compared to conventional measurement techniques, our model is well-positioned to support diagnostic and clinical applications, particularly in scenarios requiring precise blood viscosity measurement, as shown in Table 4.

One of the most notable advantages of the developed model is its minimal sample requirement. Traditional methods typically demand sample volumes upwards of several milliliters, presenting challenges in applications where sample availability is limited. In contrast, our model requires only around 1 mL of a sample while generating a minimal waste of 120 μL. This efficiency not only facilitates ease of use in clinical settings but also makes the model particularly suitable for situations where samples are costly or scarce, as in pediatric or specialized blood diagnostics.

Tailored to measure SBV and DBV specifically, our model’s capacity for low shear rate measurements makes it uniquely suited for blood-like samples. This feature enables more accurate simulation of in vivo conditions, which is critical for medical diagnostics that rely on accurate blood viscosity measurements to assess patient health. By capturing both systolic and diastolic viscosities, the model provides insights into the hemodynamic profile of patients, supporting comprehensive clinical evaluations.

Integrating the microfluidic viscosity measurement platform into the clinical environment has considerable feasibility and transformative potential [48,49]. The platform’s small volume design and low sample requirements align well with the needs of clinical laboratories, where sample conservation is often critical due to the limited availability of patient specimens. Moreover, its high-precision measurement capabilities ensure that the data obtained can contribute to reliable diagnostics, a cornerstone of clinical decision-making.

In clinical settings, diagnostic tools must meet stringent requirements for efficiency and compatibility with existing workflows [50]. This platform is expected to fill the needs by offering small-volume accurate measurements and seamless integration into established laboratory protocols. The low-cost and easy-operation program reduces the financial burden on healthcare systems and makes advanced diagnostics more accessible. The platform may support diverse clinical applications, including the monitoring of rheological properties in diseases such as diabetes and cardiovascular conditions where blood viscosity changes are indicative of disease progression [51,52]. It has the potential to be integrated into routine diagnostic panels for monitoring chronic conditions or guiding therapies by evaluating drug efficacy based on viscosity-related biomarkers.

## 4. Conclusions

Through viscosity measurements of Newtonian and non-Newtonian fluids, the concept of the resistance design method has been preliminarily validated. Chip tests demonstrated a stable, fully developed flow field, establishing a strong foundation for the chip’s ability to maintain excellent stability and accuracy when dealing with fluids of varying properties. Within the blood viscosity range of 1−10 cP, the microarray chip exhibits performance comparable to that of a rheometer. In this range, the microarray chip overcomes the physical limitations of traditional viscometers, such as unreliable low-torque measurements, small sample volume requirements, and interface artifacts. The test results for non-Newtonian fluid samples showed measured systolic blood viscosity (SBV) and diastolic blood viscosity (DBV) values of 6 cP and 11 cP, respectively. Notably, our method requires less than 5% of the sample volume compared to a rheometer, while delivering superior measurement quality. By incorporating specific shear rate designs, our in vitro vascular simulation module can accurately measure viscosity variations of analog blood under different shear rates. Clinical studies have demonstrated a correlation between blood viscosity and stroke risk. For example, traditional viscometer measurements for SBV and DBV yielded values of 4.45 cP and 13 cP, respectively. This consistency across similar viscosity measurement ranges validates the effectiveness of our proposed in vitro blood viscosity measurement model.

Furthermore, a strong correlation between stroke and the mean middle cerebral artery (mMCA) pulsatility index (PI) has been confirmed. Our system enables simultaneous monitoring of stroke and mMCA PI by analyzing correlations through blood viscosity. We anticipate that this platform can integrate additional sensors for detecting stroke-related factors, paving the way for the development of a highly specific stroke prediction platform.

## Figures and Tables

**Table 1:** The character of the Newtonian sample.

	Viscosity (cP)	Density (kg/m^3^)
Glycerin	5.21	1270
Artificial blood dry	4.9	1100

**Table 2:** The boundary conditions and explanations of geometry channel.

Function Design		Setting Condition	
α	Inlet flow zone	Flow model	Laminar flow
β	Parallel flow zone	Inlet velocity	10 (μL/min)
γ	Measurement zone	Outlet pressure	0 (Pa)
δ	Outlet flow zone	Mesh size	10–1000 (μm)
		Mesh number	272,857 (unit)
		A	Inlet of reference fluid
		B	Inlet of simulation blood
		C	Outlet of pipe geometry
		a	Width of inlet flow zone
		b	length of parallel flow zone
		c	Length of outlet flow zone

**Table 3:** Microarray parallel flow zone and flow channel’s outlet area length design (unit: mm).

Design Regions	a	b	c
Initial	6	1.29	3.5
β1	6	3	3.5
β2	6	4.5	3.5
δ1	6	1.29	7
δ2	6	4.5	7

**Table 4:** Advantages and limitations of viscosity measurement models.

Structure/ Condition	Advantage	Sample Limitation	Accuracy (%)	Functional Range (cP)	Resolution (cP)	Shear Rate (s^−1^)	Reference
Rheometer	High precision, high accuracy	Non-Newtonian fluid, ≥25 mL sample	-	1–10^7^	≥0.1	≥10^−3^	[43]
Vibration viscometer	Simple operation	Newtonian fluid, ≥20 mL sample	95%	0.4–1000	0.2	-	[44]
Magnetoelastic/Sensor	Quick, low-cost measurement	Newtonian fluid, ≥100 μL sample	-	1–10	≥0.001	-	[45]
Pressure sensor	The sensitivity was better than 0.5 cP	Non-Newtonian fluid	≥81.5%	2– 100	≥0.5	20–345.1	[46]
Microfluidic/coflowing	Simultaneously measured the multiple samples	Non-Newtonian fluid, Prepare: ≥1.5 mL	≥92%	1–10,000	≥1	1–6000	[32]
Microfluidic/coflowing	Less than 10 μL sample requirement	Non-Newtonian fluid, ≥10 μL	≥95%	1–10	≥0.1	20,000–40,000	[47]
Microfluidic/coflowing	Low shear rate, High accuracy	Non-Newtonian fluid, Prepare: ≥1 mL, Waste: 120 μL	≥95%	1–10	≥0.01	10–100	Our model

## Data Availability

The original contributions presented in this study are included in the article. Further inquiries can be directed to the corresponding author.
